# Highly Sensitive Liquid M-Z Waveguide Sensor Based on Polymer Suspended Slot Waveguide Structure

**DOI:** 10.3390/polym14193967

**Published:** 2022-09-22

**Authors:** Jiachen Han, Xihan Wu, Xuyang Ge, Yuqi Xie, Guoming Song, Lu Liu, Yunji Yi

**Affiliations:** 1 College of New Materials and New Energies, Shenzhen Technology University, Shenzhen 518118, China; 2 College of Integrated Circuits and Optoelectronic Chips, Shenzhen Technology University, Shenzhen 518118, China

**Keywords:** integrated optics, slot waveguide, polymeric Mach–Zehnder interferometer, optical sensing

## Abstract

The slot structure has great advantages in improving the sensitivity of integrated waveguide optical sensors and reducing the detection limit. We propose a polymer Mach–Zehnder interferometer (MZI) optical sensor based on the slot structure and adopted the suspended structure to improve optical field interaction with the analyte, hence boosting the sensor’s sensing accuracy. In this paper, the effects of the single waveguide width, slot width, and coupling structure of the slot waveguide on the performance of the sensor operating at a 1550 nm wavelength were analyzed. Under the premise of satisfying single-mode transmission, we designed an MZI with a branch spacing of 10 µm, arm length of 2045 µm, branch span of 700 µm, and slot region of 500 µm. The sensor’s average sensitivity was 972.1 dB/RIU, and its average detection resolution was 1.6 × 10^−6^ RIU, which is approximately 1.5 times higher than that of the suspended strip waveguide, 1.6 times higher than that of the non-suspended slot structure, and 2.1 times higher than that of the non-suspended strip waveguide.

## 1. Introduction

Sensors have been rapidly growing in importance in recent years due to their essential roles in Big Data, Internet of Things, and Artificial Intelligence. Optical sensors have a wide range of applications in science, medicine, and gas safety, owing to their high sensitivity, fast response time, and immunity to electromagnetic interference. Photonic integrated circuits (PICs) are expected to be the future direction of optical sensors. PICs have the advantages of a high chip integration level, extremely fast transmission speed, and lower energy consumption. The waveguide properties and device structure of PIC-based optical sensors are mainly studied. 

Regarding waveguide properties, waveguide structures and waveguide materials are two major research areas. Planar optical waveguide structures are widely adopted as a core sensing platform due to their mechanical stability, miniaturization, and ability to be mass produced [1]. Integrated waveguide optical sensors mainly include strip waveguides with a higher optical field interaction with the analyte than rectangular waveguides, exhibiting a better sensitivity performance. To further enhance the sensing accuracy of the sensor, the slot waveguide was proposed due to its capability of confining and guiding light in nanoscale voids (low RI) between two high-index regions [2,3]. Slot waveguides allow for higher optical field interaction with the analyte compared to other waveguides [4], and they have been employed in sensors, amplifiers, power-combiners [5], array antennas [6], and modulators [7]. Meanwhile, the slot waveguide enables light to transmit in an external medium area with a low refractive index, solving the problem of mode loss due to the principle of total internal reflection [8,9,10]. Additionally, optical waveguides can be classified into inorganic materials and organic polymer materials according to the fabrication materials [11]. In the field of inorganic materials, the high refractive index (HI) group IV semiconductor materials, such as silicon on insulators, play a major role in the development of integrated optical sensors. This is mainly due to the possibility of having strong optical field confinement and then extremely small footprint devices [12]. In 2021, Raghi S. El Shamy et al. proposed a compact optical gas sensor based on the widespread silicon-on-insulator (SOI) technology with a device sensitivity of 1070 nm/RIU and a figure-of-merit (FOM) as high as 280.8 RIU−1 at the 1.55 μm wavelength [4]. In 2022, Bingyao Shi et al. proposed a novel all-pass slot microring resonator (SMRR). The corresponding refractive index sensitivity is 403 nm/RIU, which is approximately six times greater than that of traditional microring resonator sensors [13]. Regarding the organic materials, polymers have been proven to be potential complementary candidates for inorganic PICs [14,15], they have found a wide utilization in the detection field due to their modifiability and structural designability [16]. The tunable properties of the polymer give the design and fabrication of the photonic devices much more flexibility [13]. Moreover, biocompatibility is another important and unique feature of polymers. Furthermore, polymers are compatible with three-dimensional (3D) printing, dissolution, and embossing techniques, which can reduce the preparation cost. In 2018, Xiu-You Han et al. fabricated polymer photonic integrated devices by using the UV imprinting technique [17]. In 2020, Giulia Panusa et al. demonstrated the fabrication of submicron optical waveguides in polydimethylsiloxane (PDMS) using divinylbenzene (DVB) as the photopolymerizable monomer through two-photon polymerization (2PP) [18]. Imprinting and 3D printing also can implement three-dimensional devices to improve integration and device performance. In this paper, we focused on the study of material flexibility and 3D structure, so a polymer was employed as the material to fabricate a slot waveguide. In the field of polymer sensing, strip waveguides and slot waveguides on substrates are widely employed. In 2020, Xiao Xia Ma et al. proposed and demonstrated a cost-effective liquid refractive index (RI) sensor in polymer materials by employing a Mach–Zehnder interferometer (MZI) formed with the strip waveguide [19]. Kaixin Chen et al. proposed a polymer horizontal slot waveguide as a light-analyte interactive region to implement a low-cost and highly sensitive liquid refractive index sensor [20]. To further exert the compatibility of the polymer with three-dimensional (3D) printing, dissolution, and embossing techniques, we proposed a polymer suspended slot, a three-dimensional structure that adds a test surface in contact with the test fluid, which leads to a higher sensing accuracy. The average sensitivity of the sensor we designed was 972.1 dB/RIU, and its average detection resolution is 1.6 × 10^−6^ RIU, which is approximately 1.5 times higher than that of the suspended strip waveguide, 1.6 times higher than that of the non-suspended slot structure, and 2.1 times higher than that of the non-suspended strip waveguide.

Regarding the device structure, the device structures of existing optical waveguide sensors are mainly microdisk resonators [21], microring resonators [22], MZI [23,24,25,26], and Young’s interferometer [27]. Ring resonators are compact and easy to multiplex, but an expensive and bulky high-resolution wavelength-tunable laser is often required to operate the silicon microring resonator and its detection limit is ultimately restricted by the laser resolution [28]. Additionally, in the polymer field, the bending loss of the microring is relatively large, especially for the ridge waveguide, and the optical field will leak in the slab which is not conducive to the sensing of the device. Compared with microring resonator sensors, MZI-based sensors are easier to implement and simpler regarding the design, fabrication, and measurement [28]. MZI can be divided into the symmetric structure of micro-fluidic channel integration and asymmetric without disposition according to the contact manner. In 2008, the MZI waveguide chemical sensor with an inverted ridge structure was presented by Shew et al. [29]. In 2012, Wang et al. presented an improved sensing window with three sensing surfaces, whose sensitivity could be enhanced by a factor of 2.8 in theory [30]. In 2013, Qing Liu et al. proposed a highly sensitive label-free Mach–Zehnder interferometer (MZI) biosensor based on a silicon nitride slot waveguide, and the bulk refractive index sensitivity of it was found to be 1864 π/RIU (refractive index unit) with a 7 mm-long slot waveguide sensing arm [28]. In 2021, Li, Xun et al. investigated a Compact Gas Sensor Using Silicon-on-Insulator Loop-Terminated Mach–Zehnder Interferometer, which uses a slot waveguide for the sensing arm and achieves a device sensitivity of 1070 nm/RIU [4]. In summary, MZI utilizes a sensing scheme based on optical phase shift without spectral detection, primarily measuring the output light intensity, making it more cost-effective [1]. Therefore, we adopted the MZI with a single-arm integrated slot. In the slot integration process, the coupling structure is a significant indicator affecting the coupling loss. The device needs a transition structure from the strip waveguide to slot waveguide, but the transition will cause certain energy loss. To reduce coupling loss, the design of an effective coupled structure is of great necessity. In 2011, Antao Chen et al. proposed a cooled structure with a coupling efficiency of 91.2% [31]. In this paper, to couple the polymer suspended slot, we proposed the tapers in two latitudes, which can reduce the coupling loss. 

In this paper, the potential of using a suspended slot waveguide as a light-analyte interactive region to implement a highly sensitive liquid M-Z sensor is explored. To enhance the sensing accuracy, we designed the suspended slot waveguide to expand the contact area between the sensor and the test liquid, as well as proposed the coupled structure to reduce the coupled loss. Numerical analysis reveals that with the optimized geometrical parameters, the polymer slot waveguide of a 3D structure can achieve high waveguide sensitivity in comparison with the sensors employing polymer strip waveguides and slot waveguides on substrates.

## 2. Design of the Device Structure

**Figure 1:**
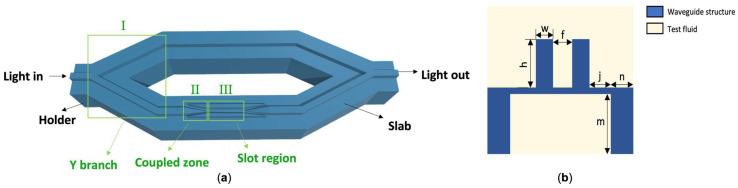
(**a**) Schematic of the liquid sensor based on the polymer suspended slot waveguide. (**b**) Cross-section of the sensor showing the holder on both sides of the waveguide and the slab beneath the device which forms the suspended structure. w, h, f, m, n, and j are the widths of waveguides on both sides of the slot, the slot height, the slot width, the holder height, the holder width, and the distance between the slot and the two holders, respectively.

The overall structure is divided into Zone I (Y branch), Zone II (coupled zone), and Zone III (slot zone). The cross-sectional view of the device is shown in Figure 1a, and the longitudinal section of the slot region is shown in Figure 1b. The device is made of polymer and is surrounded by the test liquid. The device is a suspended structure with the following dimensions: w for the waveguide width on both sides of the slot, h for the slot height, f for the slot width, m for the holder height, n for the holder width, and j for the distance between the slot and the two holders. The device was supported by brackets on both sides of the waveguide and a slab, which formed a suspended structure and realized a buried structure inside the liquid. This construction increases the contact area between the waveguide and the liquid being monitored. Meanwhile, the slot structure is used to improve the interaction between the liquid sample and the optical wave evanescent field, hence, boosting the sensor’s sensing accuracy. Based on the aforesaid structure, the device will not only be analyzed in terms of optical power ration, energy loss, and performance, but also be optimized in terms of device parameters, waveguide mode, and optical field.

## 3. Mode and Optical Field Optimization of the Slot Waveguide

**Figure 2:**
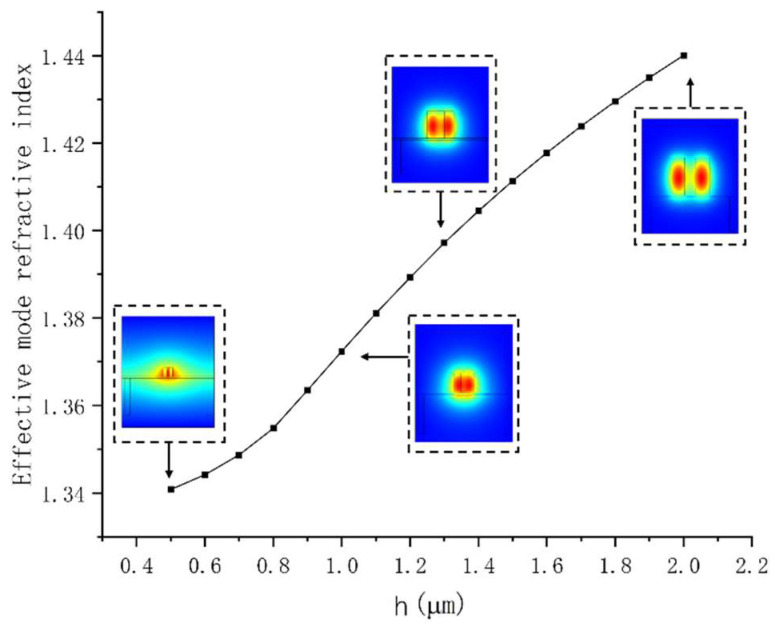
The $N_{eff}$ as functions of h for the section dimension as 2 µm × 2 µm. Insets: the distribution of the optical fields when h = 0.5 µm, h = 1.0 µm, h = 1.3 µm, and h = 2.0 µm, respectively.

The single-mode waveguide ensures the extinction ratio of the MZI and the stability of the device detection. However, to reduce the coupling loss of the device, while maintaining the compactness and fewer modes of the device, the Y-branch of the MZI adopts a size of 2 µm × 2 µm. The taper structure is used in the coupling area to compress the optical field to the slot waveguide, and the slot region needs to guarantee the single-mode characteristics of the device to realize the stability of the device detection. The slab size was kept at 100 nm to ensure machining accuracy and structural stability. The number of modes, distribution of the optical field, and mode effective refractive index ($N_{eff}$) of waveguides with varying slot cross-section sizes were simulated using the finite element method. Figure 2 depicts the relationship between waveguide size and $N_{eff}$, as well as the distribution of the optical field. As shown in Figure 2, when the section dimension of the slot waveguide is 2 µm × 2 µm, it becomes two waveguides, which cannot form a relatively high energy ratio in the slot. Meanwhile, when the section size is 0.5 µm × 0.5 µm, the optical field has a large leakage. The increase in modes is not conducive to the extinction ratio of the device. Meanwhile, when the slot waveguide’s size increases, the slab’s limitation of the slot on the light field decreases, causing light to leak from the slab. Thus, the waveguide size needs to be reduced horizontally and vertically. Nevertheless, when the waveguide is undersized, the waveguide structure is vulnerable in the process of coupling loss in the redissolving atmosphere. Finally, the slot height was set at 1 µm to ensure single-mode characteristics and low leakage loss, establishing the basic mode of the slot waveguide. The optical field distribution in Figure 2 shows that when the supports on both sides are larger than 2.2 µm, there is no effect on the optical field.

## 4. Optimization of Slot Waveguide

For the device, the sensing accuracy ($S_{w}$) depends on the slope of the phase difference and the change in the cladding refractive index. 

The waveguide sensitivity $S_{w}$ can be described as:

(1) $$S_{w}=\frac{\partial_{N_{eff}}}{\partial_{n_{c}}}$$

where $N_{eff}$ is the mode effective refractive index, $n_{c}$ is the refractive index of the upper cladding (the sensing liquid). $S_{w}$ depends on the distribution of the optical field in the test fluid, i.e., the integral share of the light in the test liquid. η represents the cladding optical field energy divided by the total energy. η is affected by the width of both the slab and the slot, which are analyzed respectively. The relationship between the integration ratio and the slot width as well as the relationship between $S_{w}$ and the slot width are analyzed for the slab widths of 0.1, 0.2, and 0.3 µm.

**Figure 3:**
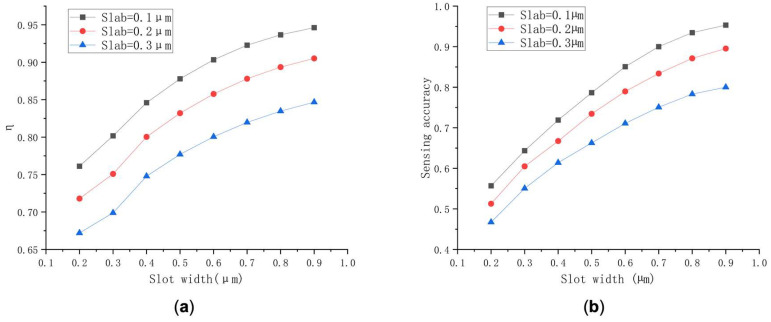
(**a**) Relationships between the value of η and the slot width with different slab sizes. (**b**) Relationships between $S_{w}\;$ and slot width with different slab sizes.

Relationships between η and slot width with different slab sizes are shown in Figure 3a, indicating that when the slot widths vary from 0.2 to 0.9 µm, the corresponding values of η for slab sizes of 0.1, 0.2, and 0.3 µm range from 0.76118 to 0.94623, 0.71795 to 0.90513, and 0.67193 to 0.84676, respectively. The value of η reaches 0.94623 when the slot width is 0.9 µm with slab thickness of 0.1 µm, surpassing that of the other two slab sizes. 

Secondly, $S_{w}$ corresponding to different structures were explored when the slot widths vary from 0.2 to 0.9 µm. The $S_{w}$ of the changed slot widths under different slab thicknesses is illustrated in Figure 3b, $S_{w}$ for slab sizes of 0.1, 0.2, and 0.3 µm varies from 0.5571 to 0.9532, 0.5127 to 0.8953, and 0.4673 to 0.8000, respectively. It can be concluded that the thinner the slab, the higher the $S_{w}$. Thus, combined with previous studies, the slab of 0.1 µm was adopted for further exploration. 

**Figure 4:**
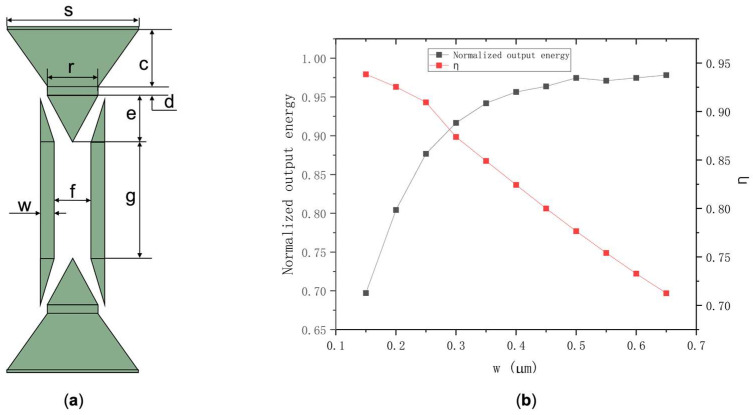
(**a**) Schematic of the coupling structure. (**b**) $I_{out}$ and η corresponding to different slot cross-section sizes.

In brief, it can be concluded that as the slot width keeps increasing, $S_{w}$ and η are rising. However, with the increase in slot width, the limitation of the optical field decreases, so the structure was adopted with a slab thickness of 0.1 µm and a slot width of 0.7 µm for further study. The increase in the value of η means the decreasing limitation of the optical field and larger coupling loss during the coupling process. Hence, the coupling structure was optimized using the finite element method for simulation, and the coupling structure we designed is illustrated in Figure 4a. Subsequently, the normalized output energy (*I_out_*) and η corresponding to the width of waveguide on both sides of slot (w in Figure 4a) were calculated. The results are shown in Figure 4b. When w is between 0.15 and 0.65 µm, the value of η varies from 0.93848 to 0.71262 and $I_{out}\;$ increase from 0.69720 to 0.97826. Consequently, it can be obtained that $S_{w}$ is positively correlated with η. To maximize device performance, taking slot parameters the posterior end of oraconaris length (s) = 2 µm, the fore part of oraconaris length (r) = 0.8 µm, the length of oraconaris (c) = 75 µm, the length of the syntaxial waveguide (d) = 25 µm, the length of coupling point (e) = 135 µm, the slot width (f) = 0.7 µm, the slot length (g) = 500 µm, and the waveguide width on both sides of the slot (w) = 0.25 µm, the normalized output energy (*I_out_*) of 0.8768 corresponds the value of η = 0.9095.

**Figure 5:**
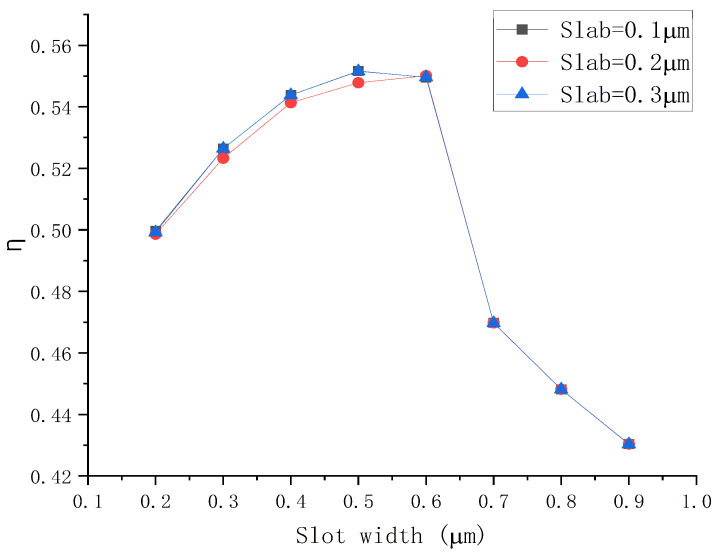
η for the device with the substrate.

In addition, we calculated η of the structure using a substrate material with a refractive index of 1.33, and the results are illustrated in Figure 5, from which it can be found that with a substrate, the maximum η can only reach up to about 55%, which is lower than that of the suspended structure.

## 5. Data Analysis and Comparison of MZI 

**Figure 6:**
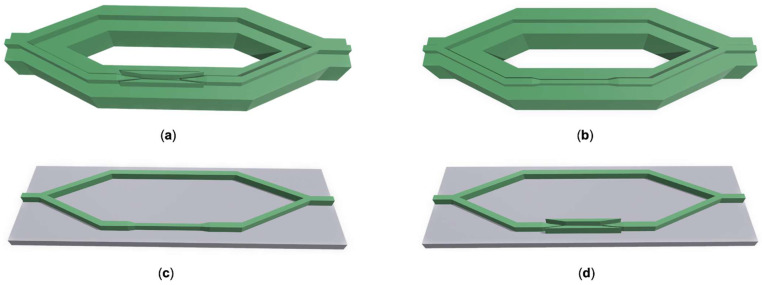
Schematics of the devices: (**a**) the suspended MZI with a slot waveguide; (**b**) the suspended MZI with a strip waveguide; (**c**) the MZI with a strip waveguide on a substrate; and (**d**) the MZI with a slot waveguide on a substrate.

The optimization analysis of the overall MZI structure was subsequently performed. The device was designed with branch interval of 10 µm, arm length of 2045 µm, and branch span of 700 µm. Furthermore, we investigated the device sensitivity ($S_{d}$) of the suspended MZI with the slot waveguide (Figure 6a), the suspended MZI with the strip waveguide (Figure 6b), the MZI with the strip waveguide on the substrate (Figure 6c), and the MZI with the slot waveguide on the substrate (Figure 6d). All their cladding is liquid, the structures in Figure 6a,d show the adopted slot waveguide, the structures in Figure 6b,c show the adopted strip waveguide. The structures on substrates with the refractive index of 1.33 are shown in Figure 6c,d. 

**Figure 7:**
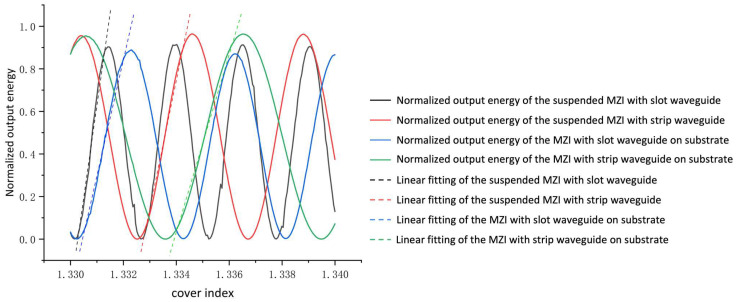
The connections between $I_{out}$ and the $n_{c}$ of the suspended MZI with a slot waveguide, the suspended MZI with a strip waveguide, the MZI with a slot waveguide on a substrate, and the MZI with a strip waveguide on a substrate.

**Figure 8:**
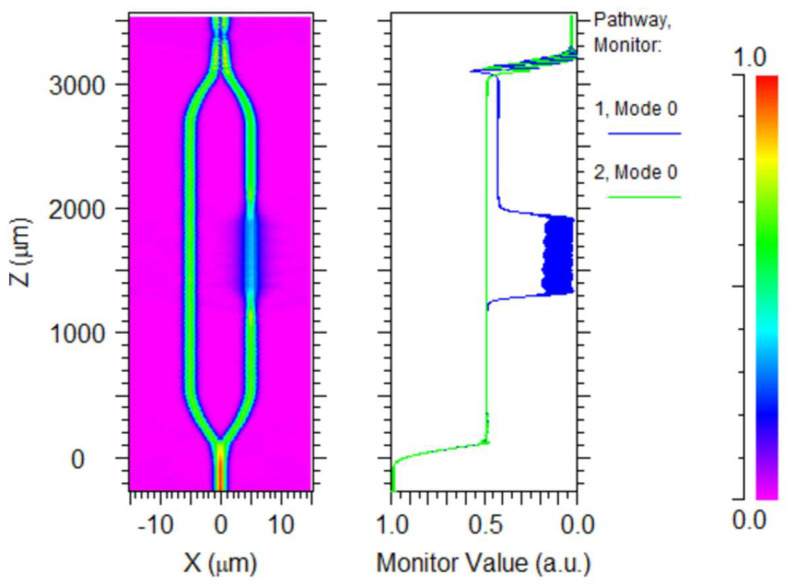
The simulated optical field of the suspended MZI with a slot.

The device sensitivity $S_{d}\;$ can be described as:

(2) $$S_{d}=\frac{\partial_{I_{out}}}{\partial_{n_{c}}}=\frac{2\pi L}{\lambda }\cdot \sin\left(\frac{2\pi L}{\lambda }\left(N_{eff1}-N_{eff2}\right)\right)\cdot \left(\frac{\partial_{N_{eff1}}}{\partial_{n_{c}}}-\frac{\partial_{N_{eff2}}}{\partial_{n_{c}}}\right)$$

where $S_{d\;}$ is the sensitivity of the sensor and $n_{c}$ is the refractive index of the test medium. *L* is the length of the branched arm, λ is the operation wavelength. $N_{eff1}\;$ and $N_{eff2}$ are the effective refractive indexes of two branched arms. We selected salt solution as the liquid analyte to be tested. When the mass fraction of the salt solution varied from 12.5% to 17.5%, the reflective index changed from 1.33 to 1.34. The schematic of different structures is shown in Figure 6, and the connections between $I_{out}$ and $n_{c}$ are illustrated in Figure 7. The suspended MZI sensor with a slot waveguide has an average sensitivity of 972.1 dB/RIU and an average detection resolution of 1.6 × 10^−6^ RIU. The suspended MZI sensor with a strip waveguide has an average sensitivity of 652.3 dB/RIU and an average detection resolution of 1.5 × 10^−6^ RIU. The average sensitivity of an MZI sensor with a strip structure on a substrate is 460.1 dB/RIU, and the average detection resolution is 2.2 × 10^−6^ RIU. The MZI sensor with a slot structure on the substrate has an average sensitivity of 601.6 dB/RIU and an average detection resolution of 1.6 × 10^−6^ RIU. It can be concluded that the average detection resolution of the suspended MZI sensor based on the slot structure we designed is about 1.5 times higher than that of the suspended strip waveguide structure, 1.6 times higher than that of the non-suspended slot structure, and 2.1 times higher than that of the non-suspended strip waveguide structure. Figure 8 illustrates the simulated optical field of different structures.

**Figure 9:**
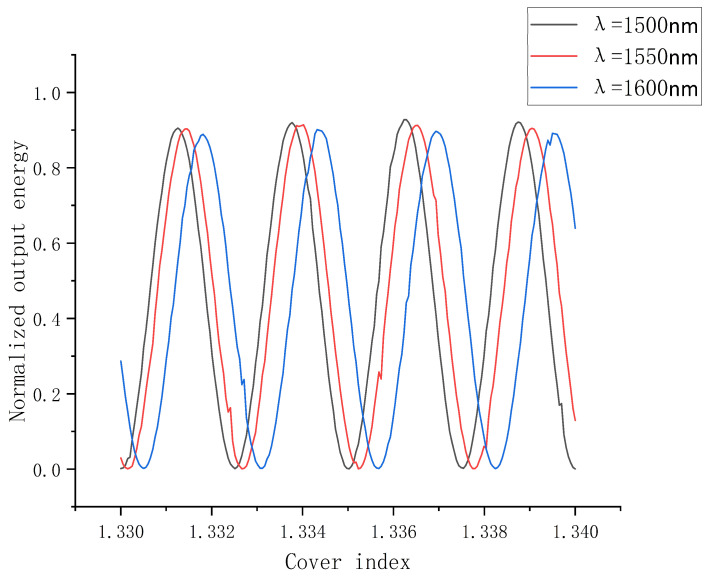
The relationship between the normalized output energy with the refractive index for the structure at wavelengths of 1500, 1550, and 1600 nm.

Regarding polymer slot waveguides, the variation in sensing accuracy with wavelength change is significantly higher than that of ridge waveguides and silicon slots. One of the main reasons is that its mode changes with wavelength, and the other is that the polymer has a high sensitivity to wavelength change owing to its low refractive index. Therefore, we analyzed the relationship between the normalized output energy with the refractive index for the structure at different wavelengths near the communication wavelength of 1550 nm. As shown in Figure 9, when the reflective index varies from 1.33 to 1.34, the corresponding $S_{d\;}$ for wavelengths of 1500, 1550, and 1600 nm are 1049.4, 972.1, and 935.9 dB/RIU, respectively. It can be concluded that the shorter wavelength will lead to better mode confinement, resulting in a higher normalized output energy. Therefore, the sensing accuracy can be enhanced by reducing the wavelength. In addition, the rapid response of polymer slots to changes in wavelength enables the polymer slot to be applied in the field of spectral detection. 

## 6. Stability Analysis of Suspended Structures

**Figure 10:**
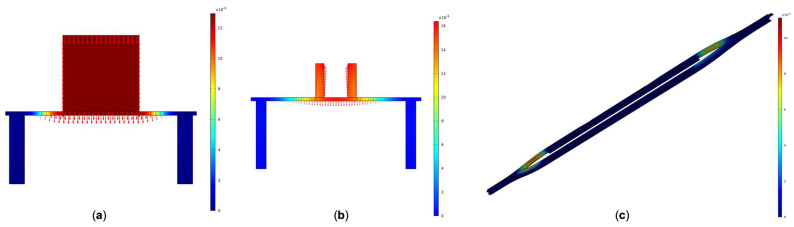
The schematic diagram of the mechanical analysis of the device: (**a**) the entry position of the central coupling region; (**b**) the slot waveguide; and (**c**) the 3D device structure.

For suspended structures, a solid support is essential to keep the device from collapsing owing to its gravity. The slabs and the holders were designed for supporting the device, but it will lead to the leakage of the light field. To ensure the stability of the device and explore the influence on the reflective index exerted by the deformation of the structure, mechanical simulation analysis was conducted on the above size of the polymer device using the finite element method. We calculated the deformation of the entry position of the central coupling region, the slot waveguide, and entire 3D device structure. The schematic diagram of the mechanical analysis of the device is shown in Figure 10. The most severe deformations of the entry position of the central coupling region (Figure 10a) and the slot waveguide (Figure 10b) are only 1.29 × 10^−4^ and 1.57 × 10^−4^ µm, proving that the optimized device has good bearing capacity and can maintain a stable suspended structure. Moreover, it can be known that the deformation of the sensing arm with a slot waveguide and the sensing arm with a straight waveguide is numerically similar, so the influence exerted by the deformation of each sensing arm on the effective refractive index is of the same order of magnitude, which can counteract each other in the calculation of the phase difference. The deformation of the 3D device structure (Figure 10c) is 0.01 µm in the Y branch region. The deformation mainly occurs at the Y branch. However, it has no effect on the phase difference and output of the device due to its symmetry. 

## 7. Conclusions

In this paper, we optimized the device parameters to enhance $I_{out}$, $S_{d}$, $S_{w}$, and η. A suspended branch arm with a slot structure that exhibits a high sensitivity was designed. The average sensitivity of the suspended MZI sensor based on the slot waveguide is 972.1 dB/RIU and the average detection resolution is 1.6 × 10^−6^ RIU, which is about 1.5 times higher than that of the suspended strip waveguide structure, 1.6 times higher than that of the non-suspended slot structure, and 2.1 times higher than that of the non-suspended strip waveguide structure. In this paper, we proposed a liquid sensor based on a polymer suspended slot waveguide compatible with imprinting and redissolving technology, which can tackle the existing problems of fabrication cost and light scattering loss of silicon slot waveguides. The liquid M-Z waveguide sensor based on the polymer suspended slot waveguide structure we proposed is expected to have great application potential in the field of liquid sensing. 

## Data Availability

The data that support the findings of this study are available from the corresponding author upon reasonable request.
